# Anxiety-Related Functional Dizziness: A Systematic Review of the Recent Evidence on Vestibular, Cognitive Behavioral, and Integrative Therapies

**DOI:** 10.3390/life16010159

**Published:** 2026-01-18

**Authors:** Rosario Ferlito, Francesco Cannistrà, Salvatore Giunta, Manuela Pennisi, Carmen Concerto, Maria S. Signorelli, Rita Bella, Maria P. Mogavero, Raffaele Ferri, Giuseppe Lanza

**Affiliations:** 1 Department of Biomedical and Biotechnological Sciences, University of Catania, 95123 Catania, Italy; ferlito.rosario@libero.it (R.F.); cannistrafrancesco02@gmail.com (F.C.); sgiunta@unict.it (S.G.); manuela.pennisi@unict.it (M.P.); 2 Department of Clinical and Experimental Medicine, University of Catania, 95123 Catania, Italy; carmen.concerto@unict.it (C.C.); maria.signorelli@unict.it (M.S.S.); 3 Department of Medical and Surgical Sciences and Advanced Technologies “G. F. Ingrassia”, University of Catania, 95123 Catania, Italy; rbella@unict.it; 4 Clinical Neurophysiology Research Unit, Sleep Research Centre, Oasi Research Institute-IRCCS, 94018 Troina, Italy; paola_mogavero@libero.it (M.P.M.); rferri@oasi.en.it (R.F.); 5 Department of Surgery and Medical-Surgical Specialties, University of Catania, 95123 Catania, Italy

**Keywords:** persistent postural-perceptual dizziness, anxiety, vestibular rehabilitation, cognitive behavioral therapy, systematic review, randomized controlled trials

## Abstract

*Background*: Functional dizziness and persistent postural-perceptual dizziness (PPPD) involve mutually reinforcing vestibular symptoms and anxiety. Non-pharmacological interventions, such as vestibular rehabilitation therapy (VRT) and cognitive behavioral therapy (CBT), aim to address both mechanisms, yet their overall effectiveness remains unclear. *Methods*: We systematically examined randomized controlled trials (RCTs) published between 2000 and 2025 that evaluated VRT, CBT, or multimodal approaches for adults with functional or chronic dizziness (including PPPD and related functional dizziness constructs) accompanied by significant anxiety. Twelve RCTs (513 participants) met the criteria, involving individuals with PPPD, chronic subjective dizziness, chronic vestibular disorders with prominent anxiety, and residual dizziness after benign paroxysmal positional vertigo. *Results*: Conventional VRT delivered in clinic or as structured home-based programs produced small-to-moderate improvements in dizziness-related disability versus usual care. Combining VRT with CBT or psychologically informed components yielded larger and more consistent reductions in disability and maladaptive dizziness-related beliefs. CBT-based interventions reduced anxiety and dizziness-related distress compared with supportive controls. Emerging modalities, including virtual-reality-based VRT, non-invasive neuromodulation, and heart-rate-variability biofeedback, showed potential, although they were limited by small samples and methodological issues. Most trials had some risk-of-bias concerns and evidence certainty ranged from very low to moderate. *Conclusions*: Integrated multimodal rehabilitation shows promise, although larger, high-quality RCTs using standardized procedures and outcome measures are required.

## 1. Introduction

Dizziness and vertigo are among the most frequent neurological and otological complaints, affecting up to one third of adults during their lifetime and exerting a substantial impact on daily functioning, quality of life, and risk of falls [1,2,3]. Beyond classic peripheral or central vestibular disorders, many patients present with persistent dizziness in the absence of ongoing structural vestibular pathology. These conditions, variously described as chronic subjective dizziness, functional dizziness, or persistent postural-perceptual dizziness (PPPD) [4,5,6], are increasingly conceptualized as disorders of multisensory integration and maladaptive postural control in which psychological factors play a key role [7].

PPPD and related functional dizziness syndromes are characterized by chronic non-spinning vertigo, unsteadiness, and dizziness, typically exacerbated by upright posture, active or passive motion, and complex or moving visual environments [5]. Patients frequently report marked anxiety, hypervigilance to bodily sensations, and avoidance of situations associated with dizziness [8]. Epidemiological studies indicate that anxiety and depressive symptoms are highly prevalent in individuals with functional dizziness and that pre-existing anxiety disorders are a strong risk factor for the development and persistence of these syndromes [6,9].

Importantly, anxiety in patients with functional dizziness is not a unitary construct. A distinction should be made between general anxiety, i.e., pre-existing or comorbid anxiety disorders characterized by pervasive worry and autonomic hyperarousal, and dizziness-related anxiety, which is specifically triggered and maintained by vestibular sensations, postural instability, or visually complex environments [10,11]. The latter is often situational and symptom-contingent, manifesting as fear of motion, imbalance, or falling, heightened visual dependence, and avoidance of dizziness-provoking contexts (such as crowded spaces) [12,13]. From an otoneurological perspective, this dizziness-specific anxiety is particularly relevant, as it directly interferes with vestibular compensation, promotes maladaptive postural strategies, and perpetuates persistent symptoms, even in the absence of ongoing peripheral vestibular pathology [13].

Neurophysiological models suggest that anxiety and dizziness interact through bidirectional loops involving vestibular nuclei, the cerebellum, brainstem autonomic centers, and cortical networks for spatial orientation, threat detection, and interoception [10,14]. According to these models, vestibular perturbations increase state anxiety and foster catastrophic interpretations of bodily sensations [6], which in turn amplify subjective dizziness and promote maladaptive behavioral responses such as stiffened posture, excessive visual dependence, and activity avoidance [8,9,15,16]. Over time, even after the initial vestibular insult has resolved, these vicious cycles can maintain persistent dizziness, functional disability, and reduced participation [15].

This conceptual framework provides a rationale for non-pharmacological interventions targeting both vestibular and psychological mechanisms. Vestibular rehabilitation therapy (VRT) promotes central vestibular compensation through habituation, adaptation, and substitution exercises [5,6,14,17], whereas cognitive behavioral therapy (CBT) and related approaches address catastrophic beliefs, avoidance patterns, and hypervigilance [6,18,19]. Recent RCTs have evaluated traditional clinic-based VRT [14,17], home-based or online programs, virtual-reality-based rehabilitation [2,4,16], psychologically informed VRT, CBT alone or combined with pharmacological treatment [6,19], neuromodulation adjuncts [20], and autonomic-oriented interventions such as heart rate variability (HRV) biofeedback [21].

Although a growing number of RCTs have examined VRT, CBT, and multimodal approaches in patients with functional or chronic dizziness, the evidence remains scattered across heterogeneous populations, interventions, and outcome measures. To our knowledge, no previous systematic review has focused specifically on non-pharmacological interventions in adults with functional or chronic dizziness and a prominent anxiety component [4], nor has any review formally appraised the certainty of evidence using the GRADE framework [22,23,24].

The primary aim of this systematic review was therefore to synthesize RCT evidence on non-pharmacological interventions, particularly VRT, CBT, and multimodal strategies, for adults with functional or chronic dizziness and relevant anxiety. Specifically, we sought to (1) evaluate the effects of these interventions on dizziness severity and dizziness-related disability; (2) examine their impact on anxiety, balance and mobility, quality of life, and falls; and (3) assess the overall certainty of the evidence and identify key gaps for future research.

## 2. Materials and Methods

### 2.1. Design and Reporting

We conducted a systematic review of randomized controlled trials with narrative synthesis. The review followed the Preferred Reporting Items for Systematic Reviews and Meta-Analyses (PRISMA) 2020 statement [25] and the methodological guidance of the Cochrane Handbook for Systematic Reviews of Interventions [26]. The protocol was not prospectively registered (e.g., in PROSPERO), and this is acknowledged as a methodological limitation in the Discussion.

### 2.2. Eligibility Criteria

Eligibility criteria were defined according to the PICO framework [27], as shown in Table 1.

Population (P): Adults (≥18 years) with functional or chronic dizziness, including PPPD, chronic subjective dizziness, chronic vestibular syndromes with a prominent anxiety component, and residual dizziness following benign paroxysmal positional vertigo (BPPV). Trials in which dizziness was secondary to acute traumatic brain injury, stroke, or other major neurological conditions were excluded.

Intervention (I): Non-pharmacological interventions designed to reduce dizziness and/or associated anxiety, including but not limited to the following:

- Vestibular rehabilitation therapy (VRT), delivered in clinic, at home or via digital/virtual-reality formats;
- Cognitive behavioral therapy (CBT) or CBT-based psychological interventions;
- Psychologically informed physiotherapy or vestibular rehabilitation;
- Multimodal programs integrating VRT with CBT or other psychological techniques;
- VRT or psychological interventions combined with neuromodulation (e.g., transcranial magnetic stimulation, transcranial direct current stimulation) or autonomic-regulation strategies (e.g., HRV biofeedback, breathing retraining).

Comparison (C): Usual care, standard VRT, pharmacological treatment alone, wait-list control, education-only interventions, or no treatment.

Outcomes (O): Primary outcomes were dizziness severity and dizziness-related disability, assessed with validated scales such as the Dizziness Handicap Inventory (DHI), Vertigo Symptom Scale, Vertigo Handicap Questionnaire, or visual analog scales for dizziness, and anxiety measured with validated instruments such as the Beck Anxiety Inventory, Hospital Anxiety and Depression Scale—Anxiety subscale, or the Hamilton Anxiety Rating Scale. Secondary outcomes included balance and mobility (e.g., Timed Up and Go test, Functional Gait Assessment), activities of daily living, health-related quality of life, falls or near-falls, and treatment acceptability and adverse events.

Only randomized controlled trials were included. Non-randomized designs, case series, single-case reports, narrative reviews, and purely pharmacological trials were excluded.

### 2.3. Information Sources and Search Strategy

We systematically searched PubMed, PEDro, Scopus, and the Cochrane Library (CENTRAL). The electronic search was performed between 1 July 2025 and 31 August 2025 and covered articles published from January 2000 to August 2025.

The search strategy combined terms related to dizziness/vertigo, anxiety, and rehabilitation using both keywords and controlled vocabulary (e.g., MeSH). An example PubMed search string was the following:

(‘dizziness’ OR ‘vertigo’ OR ‘persistent postural-perceptual dizziness’ OR ‘PPPD’ OR ‘chronic subjective dizziness’)

AND

(‘anxiety’ OR ‘panic’ OR ‘psychological’ OR ‘cognitive behavioral therapy’)

AND

(‘rehabilitation’ OR ‘vestibular rehabilitation’ OR ‘physiotherapy’ OR ‘exercise’ OR ‘virtual reality’).

Search strings were adapted for each database. Reference lists of included studies and relevant reviews were also screened to identify additional trials. Full search strategies are provided in the Supplementary Materials.

No language filters were applied at the database level; however, only full-text articles available in English were included in the final synthesis.

All records were imported into a reference manager and duplicates were removed. Titles and abstracts were screened against the eligibility criteria, followed by full-text assessment of potentially relevant articles.

Study selection was performed independently by two reviewers (Ro.F. and F.C.) under the supervision of a senior neurologist (G.L.). Discrepancies were resolved through discussion, with arbitration by G.L. when necessary.

**Figure 1:**
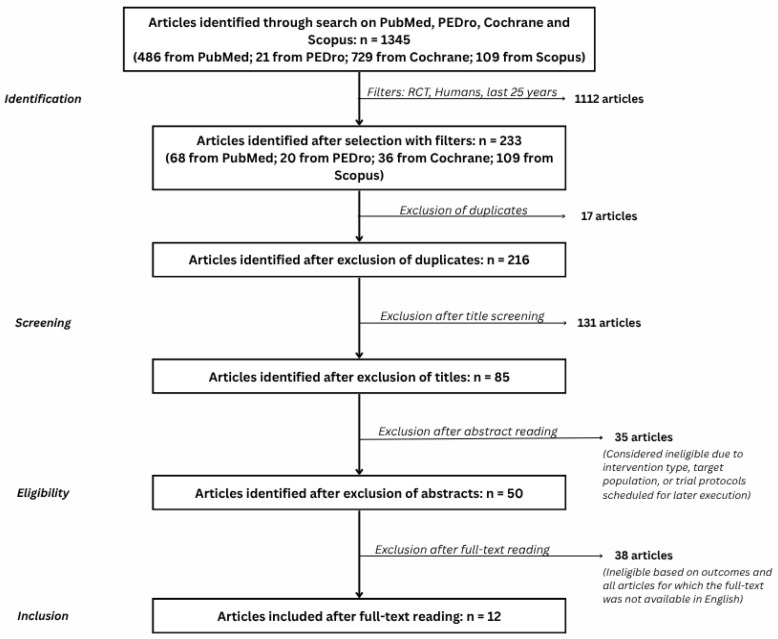
PRISMA 2020 flow diagram.

The selection process is summarized in a PRISMA 2020 flow diagram (Figure 1), including reasons for exclusion at the full-text stage.

### 2.4. Data Extraction

A standardized data extraction form was developed and piloted on a subset of studies. For each included RCT, we extracted study characteristics (first author, year, country, setting); participant characteristics (sample size, age, sex distribution, diagnostic criteria, duration of symptoms, baseline anxiety); intervention details (type of VRT and/or CBT, delivery format, dose and duration, additional components such as neuromodulation or HRV biofeedback); comparator description; outcome measures and assessment time points; main results for primary and secondary outcomes; and adverse events and drop-out rates.

Data extraction was carried out independently by two authors (Ro.F. and F.C.), with discrepancies resolved by consensus and, when required, in consultation with a third author (G.L).

### 2.5. Risk-of-Bias Assessment

Risk of bias was assessed using the Cochrane Risk of Bias 2 (RoB 2) tool for randomized trials [28,29]. The tool evaluates five domains: (1) bias arising from the randomization process; (2) bias due to deviations from intended interventions; (3) bias due to missing outcome data; (4) bias in measurement of the outcome; and (5) bias in selection of the reported result.

**Figure 2:**
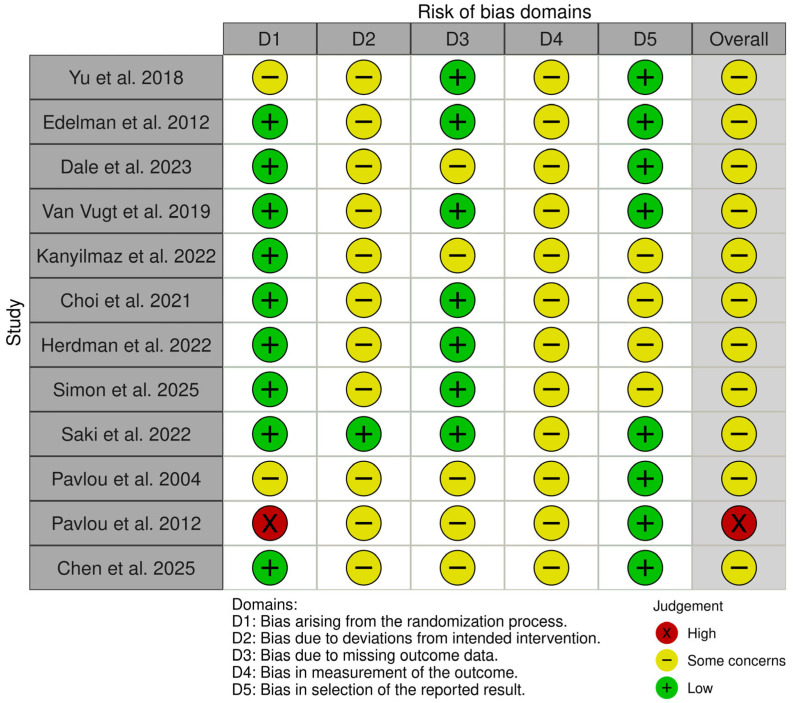
Traffic-light plot of risk of bias [2,3,4,5,6,7,8,16,17,18,20,21].

**Figure 3:**
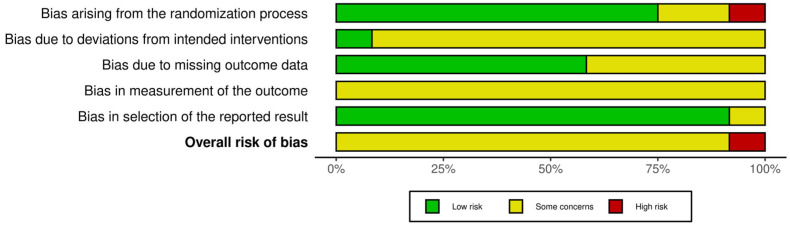
Summary plot of risk of bias.

Ro.F. and F.C. independently judged each domain as “low risk”, “some concerns”, or “high risk” and then assigned an overall risk-of-bias rating for each trial. Disagreements were resolved through discussion with G.L. as needed. Traffic-light (Figure 2) and summary plots (Figure 3) were generated to visually summarize the risk-of-bias assessments.

### 2.6. Certainty of Evidence (GRADE)

The certainty of the evidence was evaluated using the Grading of Recommendations Assessment, Development and Evaluation (GRADE) approach for key outcomes: dizziness severity/disability, anxiety, balance/mobility, and quality of life. For each outcome, certainty was rated as high, moderate, low, or very low based on risk of bias, inconsistency, indirectness, imprecision, and potential publication bias.

A Summary of Findings table, which presents effect estimates and certainty ratings for the main comparisons (e.g., VRT vs. usual care; CBT vs. control; multimodal approaches vs. standard care) and detailed GRADE assessments are both provided in the Supplementary Materials.

### 2.7. Data Synthesis

Because of marked clinical and methodological heterogeneity, including differences in diagnostic labels, intervention protocols, comparators, outcome measures, and follow-up durations, a formal meta-analysis was considered inappropriate. Instead, we performed a narrative synthesis structured by type of intervention (VRT, CBT, multimodal programs, and innovative approaches) and outcome domain.

## 3. Results

### 3.1. Study Selection

The database search yielded 1345 records: PubMed (486), PEDro (21), Scopus (109), and Cochrane (729). After removal of duplicates and application of filters for RCTs and human studies, 233 records remained for title and abstract screening. Of these, 195 were excluded as clearly irrelevant to the PICO, leaving 38 full-text articles assessed for eligibility.

Following full-text review, 26 studies were excluded for reasons such as non-randomized design, purely pharmacological interventions, inadequate outcome reporting, or absence of a relevant anxiety component. Ultimately, 12 RCTs involving 513 participants met the inclusion criteria and were included in the narrative synthesis. The study selection process is illustrated in the PRISMA flow diagram (Figure 1), whereas Table 2 shows the main characteristics of the randomized controlled trials (RCTs) included/relevant to this review.

### 3.2. Study and Participant Characteristics

The included RCTs were conducted in specialist dizziness or vestibular clinics, hospital-based rehabilitation services, and community outpatient facilities. Participants were predominantly middle-aged to older adults, with women representing most samples.

Diagnostic labels included PPPD, chronic subjective dizziness, chronic peripheral vestibulopathy with prominent anxiety, and residual dizziness after BPPV. Symptom duration ranged from several months to multiple years. Baseline anxiety was generally elevated, although the prevalence of formally diagnosed anxiety disorders varied across trials.

Sample sizes were modest, typically ranging from 30 to 80 participants per trial, and only a few studies enrolled more than 60 participants. Follow-up durations varied from immediate post-intervention assessments to 6–12-month follow-ups.

### 3.3. Interventions and Comparators

Interventions were grouped into four categories:

1. Conventional VRT: Individualized vestibular exercises (gaze stabilization, habituation, balance, and gait training) delivered in clinic or as supervised home programs. Comparators included usual medical care, minimal exercise advice, or sham interventions.
2. Psychological interventions (CBT-based): CBT protocols adapted for dizziness, focusing on catastrophic beliefs, avoidance behaviors, and misinterpretation of bodily sensations, and group psychotherapy formats targeting somatic distress and health anxiety. Comparators included wait-list control or non-specific supportive interventions, usually in addition to standard medical management.
3. Multimodal approaches: Programs combining VRT with CBT or psychologically informed counseling, and approaches integrating CBT with pharmacological treatment (e.g., sertraline) in PPPD. Comparators included pharmacological treatment alone, standard VRT, or usual care.
4. Innovative and adjunctive interventions: Virtual-reality-based VRT and optokinetic stimulation protocols; VRT combined with transcranial direct current stimulation (tDCS); and HRV biofeedback or breathing retraining aimed at autonomic regulation. Comparators typically involved standard VRT or sham neuromodulation.

Intervention duration ranged from 4 to 12 weeks, with most programs providing between 6 and 12 supervised sessions supplemented by home exercises.

### 3.4. Risk of Bias

RoB 2 assessments indicated that most trials were at a risk of bias level of “some concerns”, mainly because of limitations in allocation concealment, lack of blinding of participants and therapists (often impractical), missing outcome data, and selective reporting of secondary outcomes. One trial was judged at high risk of bias due to substantial attrition and unclear outcome reporting.

Few trials reported pre-registered protocols or detailed statistical analysis plans. Blinded outcome assessment was inconsistently implemented. Overall, the risk-of-bias profile warrants cautious interpretation of effect estimates.

### 3.5. Effects on Dizziness Severity and Disability

Dizziness severity and disability were mostly assessed by the DHI, Vertigo Symptom Scale, or analogous measures.

VRT versus usual care or minimal intervention: Conventional VRT produced statistically significant reductions in dizziness-related disability compared with control conditions in several trials. Mean differences in DHI scores generally reflected small-to-moderate improvements favoring VRT, particularly at medium-term follow-up (around 6 months). However, effect sizes varied and not all studies achieved clinically important differences on individual scales.

VRT delivered via virtual-reality or digital platforms: VR-based VRT and optokinetic stimulation produced improvements in dizziness and balance outcomes comparable to, or slightly greater than, those of conventional VRT, but sample sizes were small and follow-up durations short. Evidence remains preliminary.

Multimodal approaches (VRT + CBT or psychologically informed VRT): Trials combining vestibular exercises with CBT or psychologically informed components tended to show larger and more consistent reductions in dizziness-related disability than VRT alone. These programs were associated with greater improvements in DHI scores and reduced dizziness-related distress at follow-up.

Overall, the certainty of evidence for dizziness severity and disability ranged from low to moderate, mainly due to risk of bias, imprecision, and heterogeneity.

### 3.6. Effects on Anxiety

Anxiety was assessed using general anxiety scales (e.g., Beck Anxiety Inventory, Hospital Anxiety, and Depression Scale—Anxiety, Hamilton Anxiety Rating Scale) and, in some studies, measures of dizziness-related fear and catastrophic beliefs.

VRT alone: VRT protocols produced modest and inconsistent changes in global anxiety scores. Several trials reported improvements in dizziness-related distress and catastrophizing without corresponding large changes in general anxiety, suggesting a more specific effect on dizziness-related cognitions.

CBT and CBT-based group psychotherapy: CBT interventions, whether individual or group-based, consistently reduced anxiety scores and dizziness-related catastrophic thinking compared with wait-list or supportive control conditions. These effects were observed for both general anxiety and dizziness-specific distress.

CBT combined with pharmacological treatment: In PPPD samples, CBT plus pharmacological treatment (e.g., sertraline) yielded larger reductions in dizziness symptoms and greater treatment acceptability than pharmacological treatment alone, although the independent contribution of each component could not be disentangled.

Multimodal VRT + CBT: Psychologically informed VRT and integrated programs such as INVEST-type interventions produced meaningful reductions in both dizziness-related disability and anxiety, particularly catastrophic beliefs, and generally outperformed standard VRT on anxiety-related outcomes.

The certainty of evidence for anxiety outcomes was mostly low, reflecting small samples, variability in intervention content, and heterogeneity of measurement tools.

### 3.7. Functional Outcomes, Quality of Life and Falls

Balance and mobility were reported in a subset of trials using measures such as the Timed Up and Go test and Functional Gait Assessment. VRT-based programs generally improved these outcomes compared with usual care, and some studies suggested that VR-based and multimodal interventions might confer additional benefits. However, reporting was incomplete and follow-up periods were often short.

Quality-of-life measures and falls or near-falls were less consistently assessed. When reported, multimodal interventions tended to improve quality of life alongside dizziness and anxiety, but data were sparse and imprecise. Evidence on actual fall reduction was insufficient to draw firm conclusions.

### 3.8. Summary of GRADE Assessments

Using the GRADE framework, the certainty of evidence for the main outcomes was as follows: dizziness severity and disability, low-to-moderate certainty; anxiety, low certainty; balance and mobility, very low-to-low certainty; and quality of life and falls, very low certainty. Downgrading was primarily due to risk of bias, inconsistency of results, imprecision of effect estimates, and suspected publication bias. A Summary of Findings (Supplementary Materials) provides an overview of key comparisons and certainty ratings.

## 4. Discussion

### 4.1. Main Findings

This systematic review of 12 RCTs including 513 participants with functional or chronic dizziness and an anxiety component indicates that non-pharmacological interventions can yield small-to-moderate improvements in dizziness-related disability and, to a lesser extent, in anxiety and functional outcomes.

Conventional VRT is more effective than usual care or minimal interventions in reducing dizziness-related disability. When VRT is integrated with CBT or psychologically informed strategies, improvements in dizziness-related disability and catastrophic beliefs are larger and more consistent than with vestibular exercises alone. CBT-based interventions, whether used alone or in combination with pharmacological treatment, reduce anxiety and dizziness-related distress, although their impact on global anxiety scores is more variable. Innovative modalities such as VR-based VRT, neuromodulation adjuncts, and HRV biofeedback appear promising but remain supported by limited evidence.

Overall, these findings support the notion that multidisciplinary and multimodal approaches are more promising than single-modality treatments for addressing the intertwined vestibular and psychological mechanisms underlying functional dizziness.

From an otoneurological perspective, it is important to interpret these findings in light of the distinction between general anxiety and dizziness-related anxiety. While some patients with functional dizziness present with comorbid anxiety disorders, many exhibit anxiety that is specifically elicited by vestibular sensations, postural instability, or visually complex environments [12]. This symptom-contingent; dizziness-related anxiety plays a key role in maintaining avoidance behaviors, visual dependence, and maladaptive postural strategies, thereby interfering with vestibular compensation [13]. Our findings suggest that interventions integrating vestibular rehabilitation with psychologically informed strategies may be particularly effective because they address this dizziness-specific anxiety mechanism, rather than targeting global anxiety alone, a distinction that is highly relevant for ENT clinicians managing persistent dizziness in routine practice.

Compared with prior syntheses, this review deliberately foregrounds anxiety as a core treatment target rather than as a secondary correlate. For example, the Cochrane review of non-pharmacological interventions for PPPD prioritized vestibular symptom severity and health-related quality of life outcomes, without positioning anxiety outcomes as primary endpoints [30]. Other recent syntheses have either compared non-pharmacological treatments within PPPD more broadly or focused on vestibular physical therapy as the main active ingredient [31], while meta-analytic work on CBT has typically examined CBT as an adjunct to “conventional therapy” within PPPD cohorts [32]. Across this literature, key gaps remain: anxiety is often treated as a covariate or secondary outcome (and is not required at baseline), diagnostic labels and comparators are heterogeneous, and mechanistic integration across vestibular and cognitive behavioral domains is rarely made explicit. In contrast, we included both PPPD and earlier functional dizziness constructs (e.g., chronic subjective dizziness) when an explicit anxiety component was present, mapped interventions that directly target anxiety (CBT, counseling, relaxation/biofeedback, and exposure-based VR/optokinetic training), and graded the certainty of evidence for anxiety outcomes alongside dizziness and disability. This approach addresses a clinically relevant blind spot: anxiety is repeatedly identified as a precipitating and perpetuating factor in functional dizziness, yet it has rarely been treated as a primary endpoint in vestibular trials.

Interpretation of these findings must also consider diagnostic heterogeneity across the included trials. Although several studies explicitly enrolled patients meeting contemporary diagnostic criteria for PPPD, others relied on earlier or broader constructs, such as chronic subjective dizziness, functional dizziness, or chronic vestibular syndromes with prominent anxiety. While these entities share substantial clinical and mechanistic overlap, they are not fully interchangeable with strictly defined PPPD as per Bárány Society criteria [11]. Consequently, the applicability of pooled findings to narrowly defined PPPD populations should be interpreted with caution.

The clinical rationale for an anxiety-centered perspective is strong and arguably innovative. Functional dizziness represents a substantial clinical burden, estimated at ~10% of presentations in neuro-otology settings [12], and in chronic subjective dizziness, psychiatric disorders (predominantly anxiety) have been reported in approximately 79% of cases [33]. The Bárány Society consensus emphasizes that PPPD may be precipitated by psychological distress and involves functional alterations in postural control and multisensory processing, including cortical integration of spatial orientation with threat assessment [11]. From a mechanistic standpoint, anxiety-related hypervigilance, catastrophic appraisal, and avoidance/safety behaviors can amplify visual dependence and postural stiffening, increase autonomic arousal, and constrain vestibular compensation. Therefore, treating anxiety is not merely symptom management: it is a plausible leverage point to disrupt the anxiety–dizziness feedback loop and to improve adherence and functional recovery.

### 4.2. Interpretation Considering Pathophysiological Models

The results align with contemporary neurophysiological models in which anxiety and dizziness interact through shared neural circuits and behavioral adaptations. VRT likely exerts its effects by promoting sensory reweighting, habituation to provocative stimuli and restoration of adaptive postural strategies. CBT and psychologically informed interventions act on maladaptive cognitions, hypervigilance, and avoidance behaviors that maintain the anxiety–dizziness vicious cycle.

Multimodal interventions that combine structured vestibular exercises with CBT or targeted counseling may therefore address both sensory–motor and cognitive–emotional dimensions of functional dizziness [34]. The observed reductions in dizziness-related disability and catastrophic beliefs in these programs are consistent with this integrated mechanism [35,36].

Emerging technologies such as VR-based VRT and neuromodulation combined with VRT may further enhance sensory reweighting and neural plasticity [37,38,39], whereas autonomic-focused interventions like HRV biofeedback and breathing retraining target dysregulated autonomic arousal frequently observed in anxious dizziness. However, the evidence base for these approaches is still preliminary and insufficient to support routine implementation.

For clarity, in this review VRT refers to vestibular rehabilitation therapy, whereas VR denotes virtual reality. Conceptually, VRT and CBT likely act on complementary levels of the same neuro-vestibular network. VRT provides bottom-up, error-driven exposure to provocative head–eye movements and complex visual environments through habituation, gaze-stabilization (adaptation), substitution strategies, and balance retraining, thereby promoting sensory reweighting and reducing visual-motion sensitivity [40]. VR-based platforms can operationalize these principles with precise control of optic flow and graded complexity; randomized trials suggest that adding VR/optokinetic stimuli may be particularly beneficial for individuals with prominent visual vertigo [4] and may also reduce disability and anxiety in chronic dizziness cohorts [2]. CBT, in contrast, targets top-down processes (threat appraisal, attentional bias to bodily sensations, intolerance of uncertainty, and maladaptive avoidance), reducing anticipatory anxiety and safety behaviors that can otherwise limit the intensity and consistency of VRT exposure. When combined, CBT can facilitate engagement with symptom-provoking exercises and enhance expectancy and self-efficacy, while VRT provides experiential learning that disconfirms threat beliefs central to CBT. The INVEST approach explicitly integrates CBT principles within VRT to operationalize graded exposure and behavior change, and feasibility data support its acceptability in PPPD populations [18]. Consistent with this synergy, a meta-analysis of six RCTs reported that adding CBT to conventional therapy improved dizziness-related disability and produced statistically significant, albeit modest, reductions in anxiety measures (e.g., HAMA and GAD-7) [32].

**Figure 4:**
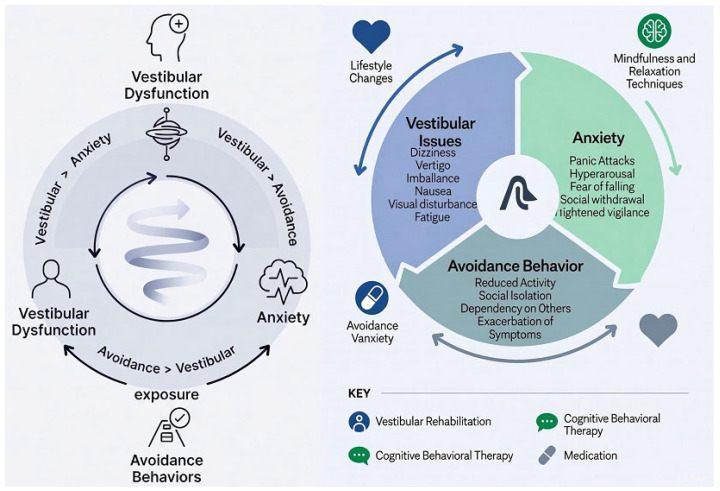
The vicious cycle of anxiety-avoidance behavior and management strategies.

Figure 4 schematically illustrates the vicious cycle of anxiety-related functional dizziness and avoidance behavior, as well as the main key strategies to manage it.

### 4.3. Clinical Implications

From a clinical standpoint, several implications emerge:

1. Systematic screening for anxiety: Patients with chronic or functional dizziness should be routinely screened for anxiety and related psychological factors using validated tools. Identifying clinically meaningful anxiety can guide referral pathways and inform the design of rehabilitation programs.
2. Integration of vestibular and psychological interventions: When an anxiety component is present, VRT alone may not be sufficient to fully address disability. Integrating CBT, psychologically informed counseling, or group psychotherapy into vestibular rehabilitation appears more likely to disrupt maladaptive cycles and support sustained improvements.
3. Role of the multidisciplinary team: Optimal management of functional dizziness with anxiety requires collaboration among otoneurologists, vestibular physiotherapists, psychologists/CBT therapists, and, when appropriate, psychiatrists. Shared treatment planning and clear communication are essential to align vestibular and psychological interventions.
4. Tailoring interventions to individual profiles: Although evidence is limited, baseline anxiety levels, catastrophic beliefs, and illness perceptions may moderate treatment response. Future clinical practice should consider these factors when selecting and sequencing VRT, CBT, and adjunctive approaches. In addition, future work should evaluate the cost-effectiveness and scalability of integrated vestibular–psychological programs, including digitally delivered components.
5. In patients with persistent or functional dizziness, identifying and targeting dizziness-related anxiety, rather than assuming a primary anxiety disorder, is crucial, as this vestibular-specific anxiety directly interferes with compensation and often requires integrated vestibular and psychologically informed rehabilitation.

### 4.4. Strengths and Limitations of the Available Evidence

The current RCT evidence has several strengths: inclusion of clinically relevant dizziness populations (PPPD, chronic subjective dizziness, chronic vestibulopathy with anxiety); evaluation of both vestibular and psychological interventions; and use of validated outcome measures across trials.

A major limitation—and a key factor underlying heterogeneity—was variability in diagnostic definitions across trials. Included studies variably enrolled patients labeled as PPPD, chronic subjective dizziness, functional or somatoform dizziness, or chronic vestibular syndromes with anxiety, often predating the Bárány Society consensus criteria for PPPD. Although these constructs overlap clinically and share common pathophysiological mechanisms (e.g., visual dependence, maladaptive postural control, anxiety-driven avoidance), they differ in operational diagnostic thresholds and symptom chronicity. Methodologically, interventions labeled as “CBT” and “VRT/VR-based rehabilitation” differed markedly in content, format, and dose (e.g., number of sessions, degree of graded exposure, home-program intensity, and type/complexity of visual-motion stimuli), and comparators ranged from usual care to multimodal programs, limiting attribution of effects to a single active component. Finally, outcomes were measured using non-uniform anxiety instruments (capturing partially different constructs) and at inconsistent time points, which complicates cross-study comparability. Collectively, these sources of heterogeneity likely explain variability in effect estimates and justify downgrading for inconsistency, and in some comparisons also for indirectness (limited generalizability to a clearly defined target population such as PPPD with clinically relevant anxiety) and imprecision (small samples and wide confidence intervals). Therefore, while the direction of effect generally favors psychologically informed and multimodal approaches, the magnitude of benefit remains uncertain and may depend on baseline anxiety severity and specific dizziness phenotypes (e.g., visual dependence/visual vertigo). As a result, the present findings cannot be assumed to apply uniformly to strictly defined PPPD populations meeting all Bárány Society criteria, and effect sizes may differ in more diagnostically homogeneous cohorts.

However, major limitations persist: small sample sizes and limited statistical power; marked heterogeneity in diagnostic criteria, intervention protocols, and follow-up durations; incomplete blinding and inadequate reporting of allocation concealment; sparse data on long-term outcomes, quality of life, and falls; and limited examination of moderators of treatment response, such as baseline psychological profiles and illness beliefs. These limitations underpin the generally low-to-moderate certainty of evidence and constrain generalizability.

### 4.5. Strengths and Limitations of the Present Review

This review has several methodological strengths. It was conducted according to PRISMA and Cochrane guidance; multiple databases were searched over a 25-year period; only RCTs were included; risk of bias was assessed systematically with RoB 2; and certainty of evidence was graded with GRADE. Study selection, data extraction, and risk-of-bias assessments were performed independently by two reviewers under the supervision of a senior researcher.

Nonetheless, some limitations must be acknowledged. The absence of protocol preregistration may increase the risk of methodological flexibility and should be addressed in future reviews. Although no language filters were applied at the search stage, only English-language full texts were included, which may have introduced language bias. The decision not to perform a meta-analysis, although justified by heterogeneity, limits the precision of pooled effect estimates. Publication bias could not be formally assessed because of the small number of trials per comparison. These issues should be considered when interpreting the findings and designing future studies.

### 4.6. Future Research Directions

Future RCTs should employ standardized diagnostic criteria for functional dizziness and PPPD; use core outcome sets including dizziness severity, anxiety, balance, quality of life, and falls; recruit larger samples with adequate statistical power and longer follow-up; clearly define and transparently report intervention content, specifying the relative contribution of vestibular, psychological, and other components; investigate moderators and mediators of treatment response, such as baseline psychological profiles, illness beliefs, and autonomic markers; and evaluate cost-effectiveness and implementation strategies for multimodal programs in real-world clinical settings.

Further trials should also attempt to disentangle the relative contribution of vestibular, cognitive behavioral, autonomic, and technology-based components within multimodal programs and determine whether baseline psychological or autonomic profiles can guide individualized rehabilitation pathways. Future RCTs should preferentially recruit patients meeting full Bárány Society diagnostic criteria for PPPD and report diagnostic stratification explicitly to determine whether treatment effects observed in broader functional dizziness populations generalize to strictly defined PPPD cohorts.

## 5. Conclusions

In adults with functional or chronic dizziness and a significant anxiety component, non-pharmacological interventions, particularly VRT and CBT, can achieve small-to-moderate reductions in dizziness-related disability. Programs that integrate vestibular rehabilitation with psychological strategies appear more promising than single-modality interventions, although the overall certainty of evidence is low-to-moderate and the RCT evidence base remains limited.

Clinicians should routinely assess anxiety and related psychological factors in patients with persistent dizziness and, when appropriate, adopt a multidisciplinary, multimodal approach. Further high-quality RCTs are needed to refine and validate integrated rehabilitation protocols, clarify the role of innovative technologies and autonomic-focused interventions, and determine how best to individualize treatment in this complex and burdensome condition.

## Figures and Tables

**Table 1:** PICO framework for the clinical question (non-pharmacological interventions for functional/chronic dizziness with anxiety).

PICO Element	Summary
Population	Adults (≥18 years) with functional or chronic dizziness where anxiety/threat appraisal is clinically relevant (e.g., PPPD, chronic subjective/functional dizziness). Typical exclusions: acute/unstable neurological disease, severe medical instability, inability to participate in rehabilitation/psychological treatment.
Intervention	Non-pharmacological interventions targeting dizziness and/or anxiety, typically delivered over 4–12 weeks (often 6–12 supervised sessions plus home practice). Examples: vestibular rehabilitation therapy (VRT), cognitive behavioral therapy (CBT), psychologically informed VRT, integrated VRT + CBT programs, virtual-reality/optokinetic exposure, and adjuncts such as HRV biofeedback or neuromodulation combined with VRT.
Comparison	Usual care, standard VRT, pharmacological treatment alone, wait-list/no treatment, education-only, sham neuromodulation, or alternative active interventions. Comparators are chosen to estimate the added value beyond baseline care.
Outcomes	Primary: dizziness severity/disability (e.g., DHI, Vertigo Symptom Scale) and anxiety (e.g., HADS-A, BAI, GAD-7, HARS). Secondary: balance/mobility (e.g., TUG, DGI), avoidance/safety behaviors, depression, quality of life, falls/near-falls, adherence/acceptability, and adverse events. Measurement time points commonly include baseline, post-intervention, and follow-up (e.g., 3–12 months) when available.

**Table 2:** Characteristics of the randomized controlled trials (RCTs) included/relevant to this review.

RCT (Population)	Design	Sample Size	Randomization	Blinding	Follow-Up	Key Results (Dizziness/Anxiety)
Yu et al. 2018 [5] (PPPD)	Parallel	CBT + sertraline (*n* = 46) vs. sertraline (*n* = 45); total N = 91	NR (reported as randomized assignment)	NR (behavioral + drug); likely open-label	8 weeks (baseline, wk2, wk4, wk8)	Greater improvement with CBT + sertraline vs. sertraline alone on DHI and anxiety scale; fewer adverse events reported.
Edelman et al. 2012 [6] (chronic subjective dizziness)	Parallel	Immediate CBT vs. wait-list; total N = 41	NR	None/NR	Post-treatment after 3 sessions (weekly)	Reduced dizziness disability; psychological outcomes on DASS not significantly changed.
Dale et al. 2023 [7] (functional/somatoform dizziness)	Parallel	IPGT + care-as-usual vs. self-help group + care-as-usual; total N = 174	Computer-generated; blocked; stratified by center; independent allocation	Outcome assessors blinded; participants/therapists not blinded	Post-treatment (16 weeks) + 12-month follow-up	IPGT produced small-to-moderate improvements in vertigo-related disability; some effects sustained at 12 months.
Herdman et al. 2022 [18] INVEST (vestibular symptoms with psychological component)	Parallel (feasibility)	Intervention vs. current guideline care; total N = 40	NR (reported; feasibility allocation)	None/NR	Feasibility follow-up (short-term) + planned longer follow-up	Small-to-moderate improvements in dizziness severity and quality of life; feasibility outcomes (acceptability, adherence).
van Vugt et al. 2019 [3] (chronic vestibular syndrome; general practice)	Parallel, 3-arm	Internet-based VRT vs. stand-alone booklet vs. usual care (N NR here)	NR	None/NR	3–6 months	Internet-based VRT showed small improvements in dizziness at 3 and 6 months vs. usual care in synthesis.
Choi et al. 2021 [4] (PPPD)	Parallel	Vestibular exercise +/− optokinetic/VR exposure; N NR here	NR	None/NR	Post-treatment; short follow-up	Both groups improved; between-group differences on functional measures often non-significant.
Pavlou et al. 2004 [8] (refractory dizziness/visual vertigo)	Parallel	Exposure-based (simulator/visual motion) rehab vs. control; N NR here	NR	None/NR	Long-term follow-up reported	Sustained improvements in visually induced dizziness reported in long-term follow-up.
Johansson et al. 2001 [19] (older adults with dizziness)	Parallel	VRT +/− CBT components; N NR here	NR	None/NR	NR	Early evidence supporting added benefit of CBT-style strategies for disability/avoidance in selected patients.
Saki et al. 2022 [20] (chronic vestibular dysfunction)	Parallel	VRT + tDCS vs. VRT + sham; N NR here	NR	Double-blind (tDCS vs. sham)/NR for other elements	NR	Improvements reported in dizziness and related outcomes with active tDCS adjunct; detailed effects NR here.

## Data Availability

No new data were created or analyzed in this study.

## Supplementary Materials

The following supporting information can be downloaded at https://www.mdpi.com/article/10.3390/life16010159/s1, Figure S1: full search strategies; Table S1: GRADE evidence profile (CI: confidence interval; MD: mean difference).

## Abbreviations

The following abbreviations are used in this manuscript:

BAI	Beck Anxiety Inventory
BPPV	Benign Paroxysmal Positional Vertigo
CBT	Cognitive Behavioral Therapy
CENTRAL	Cochrane Central Register of Controlled Trials
DASS	Depression Anxiety Stress Scales
DGI	Dynamic Gait Index
DHI	Dizziness Handicap Inventory
GRADE	Grading of Recommendations Assessment, Development and Evaluation
HADS	Hospital Anxiety and Depression Scale
HADS-A	Hospital Anxiety and Depression Scale—Anxiety subscale
HARS	Hamilton Anxiety Rating Scale
HRV	Heart Rate Variability
IPGT	Integrative Psychodynamic Group Therapy
INVEST	Integrated Vestibular and Cognitive Behavioral Therapy (trial/program name)
MeSH	Medical Subject Headings
NR	Not Reported
PICO	Population, Intervention, Comparison, Outcomes
PPPD	Persistent Postural-Perceptual Dizziness
PRISMA	Preferred Reporting Items for Systematic Reviews and Meta-Analyses
RCT	Randomized Controlled Trial
RoB 2	Risk of Bias 2 tool
tDCS	Transcranial Direct Current Stimulation
TUG	Timed Up and Go test
VR	Virtual Reality
VRT	Vestibular Rehabilitation Therapy
