# Supplementary material for: Anxiety-Related Functional Dizziness: A Systematic Review of the Recent Evidence on Vestibular, Cognitive Behavioral, and Integrative Therapies
Source: Life (Basel). 2026 Jan 18;16(1):159. doi: 10.3390/life16010159 (PMC12842955; doi:10.3390/life16010159)
Supplement: Supplementary file 1 [file life-16-00159-s001.zip › life-4101602-supplementary.pdf]

Supplementary Material

Figure S1: full search strategies.

DATABASE	SEARCH STRING
Pubmed	(Vertigo[Mesh] OR Dizziness[Mesh] OR Vestibular Diseases[Mesh] OR vertigo[tiab] OR dizziness[tiab] OR "vestibular symptom*"[tiab] OR "chronic dizziness"[tiab] OR "psychogenic dizziness"[tiab] OR "functional dizziness"[tiab] OR "visual vertigo"[tiab] OR "visually induced dizziness"[tiab] OR ((space[tiab] OR visually[tiab]) AND motion[tiab] AND (discomfort[tiab] OR sensitivity[tiab])) OR "chronic subjective dizziness"[tiab] OR "phobic postural vertigo"[tiab] OR "persistent postural-perceptual dizziness"[tiab] OR PPPD[tiab]) AND (Anxiety[Mesh] OR Anxiety Disorders[Mesh] OR Panic Disorder[Mesh] OR Panic[Mesh] OR anxiety[tiab] OR anxious[tiab] OR "panic disorder"[tiab] OR "panic attack*"[tiab] OR "Stress, Psychological"[Mesh] OR "psychological stress"[tiab] OR Psychological Distress[Mesh] OR distress[tiab] OR Activities of Daily Living[tiab] OR "fear avoidance"[tiab] OR "fear-avoidance"[tiab]) AND (Physical Therapy Modalities[Mesh] OR Exercise Therapy[Mesh] OR Rehabilitation[Mesh] OR Cognitive Behavioral Therapy[Mesh] OR Psychotherapy[Mesh] OR Exposure Therapy[Mesh] OR Relaxation Therapy[Mesh] OR "Biofeedback, Psychology"[Mesh] OR Breathing Exercises[Mesh] OR Virtual Reality[Mesh] OR "vestibular rehabilitation"[tiab] OR "vestibular therapy"[tiab] OR "gaze stabiliz*"[tiab] OR habituation[tiab] OR "balance training"[tiab] OR "postural control"[tiab] OR physiotherapy[tiab] OR "combined modality therapy"[Mesh] OR "combined therap*"[tiab] OR multidisciplinary[tiab] OR multimodal[tiab] OR "anxiety management"[tiab] OR CBT[tiab]) NOT (Anti-Anxiety Agents[Mesh] OR Antidepressive Agents[Mesh] OR Benzodiazepines[Mesh] OR Serotonin Uptake Inhibitors[Mesh] OR pharmacolog*[tiab] OR medication*[tiab] OR drug*[tiab])
PEDro	Abstract & title: vertigo AND anxiety; Published since: 2000.
Scopus	(vertigo OR dizziness) AND (anxiety OR anxiety disorder OR panic disorder OR activities of daily living OR fear avoidance) AND (physical therapy modalities OR rehabilitation OR cognitive behavioral therapy OR psychotherapy OR vestibular rehabilitation OR vestibular therapy OR physiotherapy OR CBT) AND NOT (serotonin uptake inhibitor* OR pharmacolog* OR medication* OR drug*) AND (randomized controlled trial)
Cochrane library	(Vertigo OR Dizziness OR Vestibular Diseases OR vertigo OR dizziness OR "vestibular symptom" OR "chronic dizziness" OR "psychogenic dizziness" OR "functional dizziness" OR "visual vertigo" OR "visually induced dizziness" OR ((space OR visually) AND motion AND (discomfort OR sensitivity)) OR "chronic subjective dizziness" OR "phobic postural vertigo" OR "persistent postural-perceptual dizziness" OR PPPD) AND (Anxiety OR Anxiety Disorders OR Panic Disorder OR Panic OR anxiety OR anxious OR "panic disorder" OR "panic attack" OR "Stress, Psychological" OR "psychological stress" OR Psychological Distress OR distress OR Activities of Daily Living OR "fear avoidance" OR "fear-avoidance") AND (Physical Therapy Modalities OR Exercise Therapy OR Rehabilitation OR Cognitive Behavioral Therapy OR Psychotherapy OR Exposure Therapy OR Relaxation Therapy OR "Biofeedback, Psychology" OR Breathing Exercises OR Virtual Reality OR "vestibular rehabilitation" OR "vestibular therapy" OR "gaze stabiliz" OR habituation OR "balance training" OR "postural control" OR physiotherapy OR "combined modality therapy" OR "combined therap" OR multidisciplinary OR multimodal OR "anxiety management" OR CBT) NOT (Anti-Anxiety Agents OR Antidepressive Agents OR Benzodiazepines OR Serotonin Uptake Inhibitors OR pharmacolog OR medication OR drug) AND (RCT OR randomized controlled trial)

Table S1: GRADE evidence profile (CI: confidence interval; MD: mean difference).

Question: The non-pharmacological intervention (VRT, CBT, VR-Imm, combined approaches) compared to usual care, standard VRT, or no treatment for adults with chronic or functional dizziness with an anxious component.

Setting: Physiotherapy clinics, specialist clinics, hospital, and community rehabilitation settings

Certainty assessment							№ of patients		Effect		Certainty	Importance
№ of studies	Study design	Risk of bias	Inconsistency	Indirectness	Imprecision	Other considerations	Non-pharmacological intervention (VRT, CBT, VR-Imm, combined approaches)	usual care, standard VRT, or no treatment	Relative (95% CI)	Absolute (95% CI)		
Severity of Dizziness Symptoms (assessed with: DHI/VSS/VVAS/VHQ)												
10	randomized trials	not serious	serious	not serious	serious[a]		Most studies showed a clinically relevant reduction in the Dizziness Handicap Inventory (DHI), with mean values ranging from –8 to –15 points. Van Vugt et al. (2019) reported a mean reduction of –4.6 points (95% CI –8.2 to –1.1) at 3 months and –4.9 points (95% CI –8.4 to –1.3) at 6 months. In the INVEST RCT (Herdman et al. 2022), the estimated effect of the integrated CBT-VRT intervention was SMD 0.45 (95% CI –0.12 to 1.02), with the CI crossing zero indicating uncertainty. Not all studies reported CIs: some only present means ± standard deviation, with significance based on p-value.				-[a]	CRITICAL
Anxiety (assessed with: BAI / HADS-A / HARS)												

Certainty assessment							№ of patients		Effect		Certainty	Importance
№ of studies	Study design	Risk of bias	Inconsistency	Indirectness	Imprecision	Other considerations	Non-pharmacological intervention (VRT, CBT, VR-Imm, combined approaches)	usual care, standard VRT, or no treatment	Relative (95% CI)	Absolute (95% CI)		
9	randomized trials	serious[b]	serious[c]	not serious	serious[d]		The mean reduction in scores ranged between −4 and −7 points on the BAI and approximately −2 points on the HADS-A. In some studies (e.g., Choi 2021, Simon 2025), no significant differences emerged compared to control. Variability between studies, without published CIs.				-[b,c,d]	CRITICAL
Functional Disability and ADL (assessed with: TUG/ADL)												
3	randomized trials	not serious	not serious	not serious	serious[e]		Choi et al. (2021) reported improvements in ADL and TUG (≈1–2 seconds) in both groups with no significant differences. Kanyılmaz et al. (2022) showed a superior effect of immersive VR compared to conventional VRT, maintained at 6 months. Pavlou et al. (2004) documented functional improvements in both groups, with greater benefit from visual exposure and maintenance up to 36 months.				-[e]	IMPORTANT
Quality of Life (assessed with: HRQoL: EQ-5D-5L/EQ-VAS; Scale from: 0 to 1)												

1[f]	randomized trials	serious[g]	not serious	not serious	serious[h]		20	20	-	MD 0.06 lower (0.19 lower to 0.07 higher)	-[g,h]	IMPORTANT
Risk of Falls (assessed with: DGI/FES-I/TUG)												
2	randomized trials	serious[i]	not serious	not serious	serious[j]		Kanyılmaz et al. (2022) showed that immersive VRT is more effective than conventional VRT on the TUG (−1.5/−2 sec) and DGI (+2/3 points), with effects maintained at 6 months. Pavlou et al. (2004) documented a greater improvement with visual exposure/simulators compared to standard exercises, maintained long-term (16–36 months).				-[i,j]	IMPORTANT

Explanations

a. CIs not reported in multiple studies and small sample sizes in some RCTs; possible uncertainty regarding the magnitude of the effect.

b. Self-reported outcomes, lack of blinding in almost all studies.

c. Heterogeneity between studies; some show marked reduction (e.g., Yu 2018), others minimal changes (e.g., Choi 2021).

d. Small sample sizes; CIs not always reported; in some studies, effects include values close to no change.

e. Small sample sizes (Total N ~200); CIs not reported in several studies; limited follow-up; lack of blinding.

f. Only the INVEST RCT reports QoL with standard measures (EQ-5D-5L, EQ-VAS).

g. Single-site study, intervention therapist involved in development; self-reported outcomes; impossibility of blinding; possible performance/measurement bias.

h. Small sample size (Total N=40) and CIs that include the “no effect” for EQ-5D and EQ-VAS.

i. Lack of blinding in both studies; partly subjective outcomes (FES-I) and small sample sizes.

j. Very small sample sizes (Overall N <150), CIs not reported; potentially fragile results.

Non-pharmacological intervention (VRT, CBT, VR-Imm, combined approaches) compared to usual care, standard VRT, or no treatment for adults with chronic or functional dizziness with an anxious component.

Patient or population: adults with chronic or functional dizziness with an anxious component.

Setting: Physiotherapy clinics, specialist clinics, hospital, and community rehabilitation settings

Intervention: The non-pharmacological intervention (VRT, CBT, VR-Imm, combined approaches)

Comparison: usual care, standard VRT, or no treatment

Outcomes	Anticipated absolute effects* (95% CI)		Relative effect (95% CI)	№ of participants (studies)	Certainty of the evidence (GRADE)	Comments
	**Risk with usual care, standard VRT, or no treatment**	**Risk with the non-pharmacological intervention (VRT, CBT, VR-Imm, combined approaches)**				
Severity of Dizziness Symptoms assessed with: DHI/VSS/VVAS/VHQ	Most studies showed a clinically relevant reduction in the Dizziness Handicap Inventory (DHI), with mean values ranging from –8 to –15 points. Van Vugt et al. (2019) reported a mean reduction of –4.6 points (95% CI –8.2 to –1.1) at 3 months and –4.9 points (95% CI –8.4 to –1.3) at 6 months. In the INVEST RCT (Herdman et al. 2022), the estimated effect of the integrated CBT-VRT intervention was SMD 0.45 (95% CI –0.12 to 1.02), with the CI crossing zero indicating uncertainty. Not all studies reported CIs: some only present means ± standard deviation, with significance based on p-value.			(10 RCTs)	-[a]	

Non-pharmacological intervention (VRT, CBT, VR-Imm, combined approaches) compared to usual care, standard VRT, or no treatment for adults with chronic or functional dizziness with an anxious component.

Patient or population: adults with chronic or functional dizziness with an anxious component.

Setting: Physiotherapy clinics, specialist clinics, hospital, and community rehabilitation settings

Intervention: The non-pharmacological intervention (VRT, CBT, VR-Imm, combined approaches)

Comparison: usual care, standard VRT, or no treatment

Outcomes	**Anticipated absolute effects*** (95% CI)		Relative effect (95% CI)	№ of participants (studies)	Certainty of the evidence (GRADE)	Comments
	**Risk with usual care, standard VRT, or no treatment**	**Risk with the non-pharmacological intervention (VRT, CBT, VR-Imm, combined approaches)**				
Anxiety assessed with: BAI / HADS-A / HARS	The mean reduction in scores ranged between −4 and −7 points on the BAI and approximately −2 points on the HADS-A. In some studies (e.g., Choi 2021, Simon 2025), no significant differences emerged compared to control. Variability between studies, without published CIs.			(9 RCTs)	-[b,c,d]	
Functional Disability and ADL assessed with: TUG/ADL	Choi et al. (2021) reported improvements in ADL and TUG (≈1–2 seconds) in both groups with no significant differences. Kanyılmaz et al. (2022) showed a superior effect of immersive VR compared to conventional VRT, maintained at 6 months. Pavlou et al. (2004) documented functional improvements in both groups, with greater benefit from visual exposure and maintenance up to 36 months.			(3 RCTs)	-[e]	

Non-pharmacological intervention (VRT, CBT, VR-Imm, combined approaches) compared to usual care, standard VRT, or no treatment for adults with chronic or functional dizziness with an anxious component.

Patient or population: adults with chronic or functional dizziness with an anxious component.

Setting: Physiotherapy clinics, specialist clinics, hospital, and community rehabilitation settings

Intervention: The non-pharmacological intervention (VRT, CBT, VR-Imm, combined approaches)

Comparison: usual care, standard VRT, or no treatment

Outcomes	Anticipated absolute effects* (95% CI)		Relative effect (95% CI)	№ of participants (studies)	Certainty of the evidence (GRADE)	Comments
	**Risk with usual care, standard VRT, or no treatment**	**Risk with the non-pharmacological intervention (VRT, CBT, VR-Imm, combined approaches)**				
Quality of Life assessed with: HRQoL: EQ-5D-5L/EQ-VAS Scale from: 0 to 1	The mean quality of Life was 0	MD 0.06 lower (0.19 lower to 0.07 higher)	-	40 (1 RCT)[f]	-[g,h]	
Risk of Falls assessed with: DGI/FES-I/TUG	Kanyılmaz et al. (2022) showed that immersive VRT is more effective than conventional VRT on the TUG (−1.5/−2 sec) and DGI (+2/3 points), with effects maintained at 6 months. Pavlou et al. (2004) documented a greater improvement with visual exposure/simulators compared to standard exercises, maintained long-term (16–36 months).			(2 RCTs)	-[i,j]	

*The risk in the intervention group (and its 95% confidence interval) is based on the assumed risk in the comparison group and the relative effect of the intervention (and its 95% CI).

Explanations

a. CIs not reported in multiple studies and small sample sizes in some RCTs; possible uncertainty regarding the magnitude of the effect.

b. Self-reported outcomes, lack of blinding in almost all studies.

c. Heterogeneity between studies; some show marked reduction (e.g., Yu 2018), others minimal changes (e.g., Choi 2021).

d. Small sample sizes; CIs not always reported; in some studies, effects include values close to no change.

e. Small sample sizes (Total N ~200); CIs not reported in several studies; limited follow-up; lack of blinding.

f. Only the INVEST RCT reports QoL with standard measures (EQ-5D-5L, EQ-VAS).

g. Single-site study, intervention therapist involved in development; self-reported outcomes; impossibility of blinding; possible performance/measurement bias.

h. Small sample size (Total N=40) and CIs that include the "no effect" for EQ-5D and EQ-VAS.

i. Lack of blinding in both studies; partly subjective outcomes (FES-I) and small sample sizes.

j. Very small sample sizes (Overall N <150), CIs not reported; potentially fragile results.

GRADE Working Group grades of evidence

High certainty: we are very confident that the true effect lies close to that of the estimate of the effect.

Moderate certainty: we are moderately confident in the effect estimate: the true effect is likely to be close to the estimate of the effect, but there is a possibility that it is substantially different.

Low certainty: our confidence in the effect estimate is limited: the true effect may be substantially different from the estimate of the effect.

Very low certainty: we have very little confidence in the effect estimate: the true effect is likely to be substantially different from the estimate of effect.
